# The result of desensitization of children with severe Ig-E mediated cow’s milk allergy

**DOI:** 10.1186/2045-7022-1-S1-P106

**Published:** 2011-08-12

**Authors:** Silviva Novakova, Ivan Novakov, Manuela Joncheva

**Affiliations:** 1 University Hospital, Plovdiv, Bulgaria

## Background

Our aim is to demonstrate the success of desensitization in children with severe Ig-E mediated cow's milk allergy.

## Materials and methods

10 children from 6 to 10 year of age were desensitize by introducing increasing doses of cow milk (CM) till 200 ml or the highest tolerated quality for approximally 6 months. Ig-E mediated cow’s milk allergy was convinced by skin prick test and/or specific IgE. Double – blind placebo controlled food challenge has confirmed the diagnosis. All children were observed on the first and the third months after concluding the desensitization.

## Results

Eight of the children (80%) successfully achieved the amount of 200 ml of CM daily in six months, with no adverse reactions. One of the children (10%) tolerated 32 ml and another one (10%) reached to 128 ml. All children did not present any symptoms, taking whole CM even on the third month.

## Conclusions

We successfully desensitized 8 of 10 children and induced tolerance to a smaller amount of CM in another 2. The protocol is well tolerated and results are reliable.

